# Supplementary material for: Stabilization of CDK6 by ribosomal protein uS7, a target protein of the natural product fucoxanthinol
Source: Commun Biol. 2022 Jun 9;5:564. doi: 10.1038/s42003-022-03522-6 (PMC9184650; doi:10.1038/s42003-022-03522-6)
Supplement: Supplementary file 2 — Supplementary Information file [file 42003_2022_3522_MOESM2_ESM.pdf]

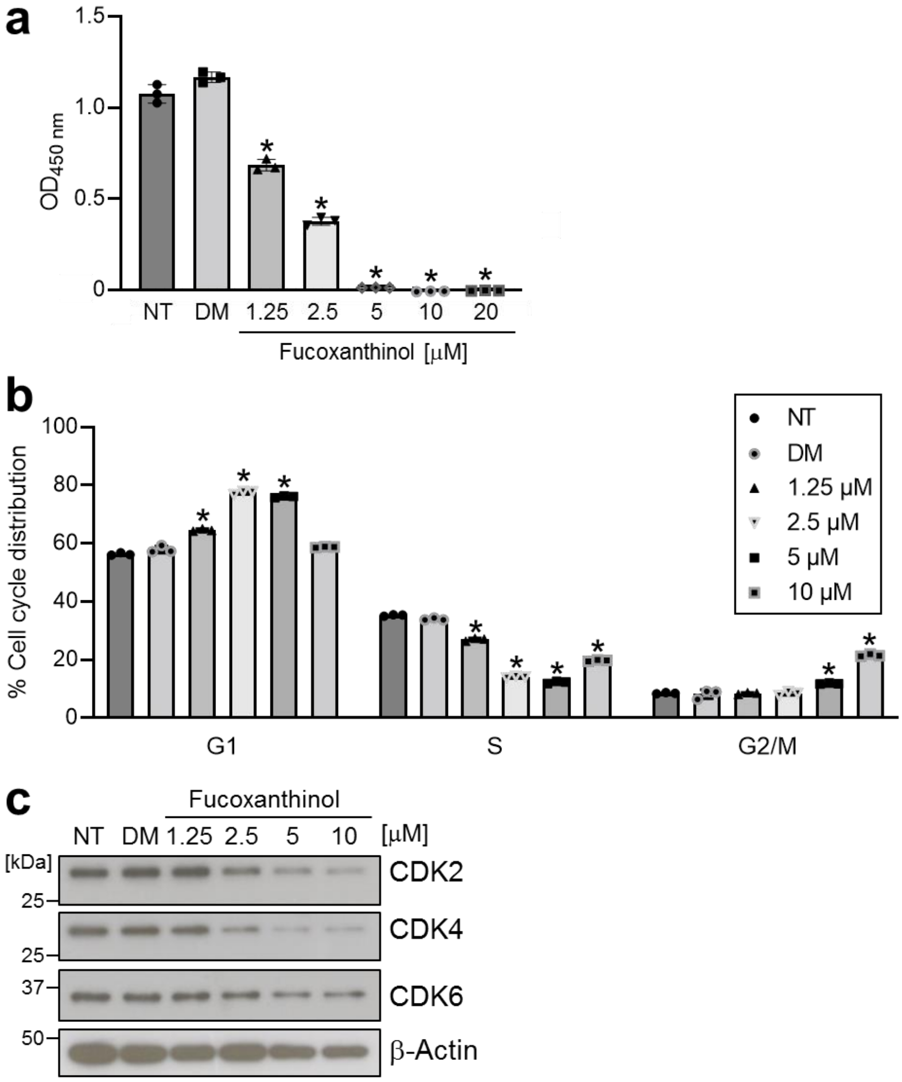

Supplementary Fig. 1 Fucoxanthinol induces G1 cell cycle arrest with the downregulation of CDK2, 4, and 6 protein expression in SW480 cells. (**a**) Human colon cancer SW480 cells were treated with the indicated concentrations of fucoxanthinol for 72 h. Cell proliferation was measured using the CCK-8 assay. (**b**) SW480 cells were treated with fucoxanthinol for 24 h, and the cell cycle was analyzed by flow cytometry. (**c**) Western blot analysis of SW480 cells treated with fucoxanthinol for 24 h. NT: non-treated, DM: 0.1% DMSO, Data are means ± S.D. (n = 3 biologically independent experiments). *$P < 0.05$ significantly different from DM.

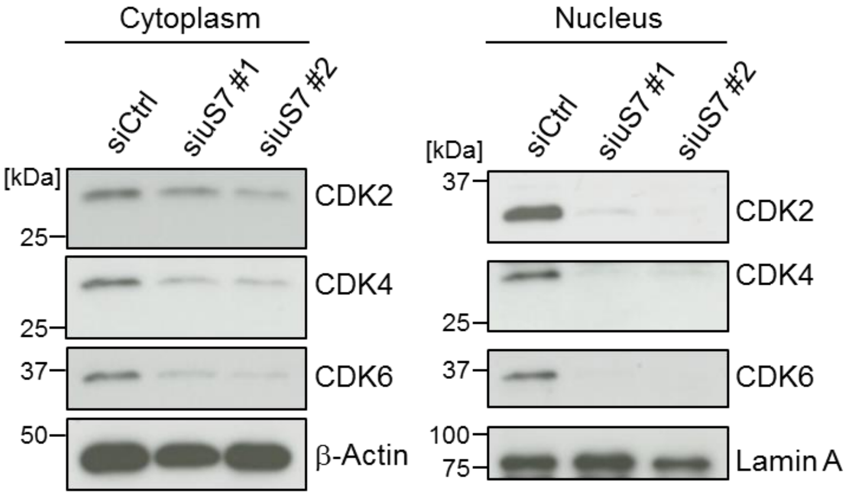

Supplementary Fig. 2 The depletion of uS7 downregulates CDK2, 4, and 6 protein expression in both the cytoplasm and nucleus. HT-29 cells treated with siCtrl, siuS7 #1, or siuS7 #2 for 48 h were lysed and fractionated into cytoplasmic and nuclear fractions. Protein expression levels were examined by Western blotting.

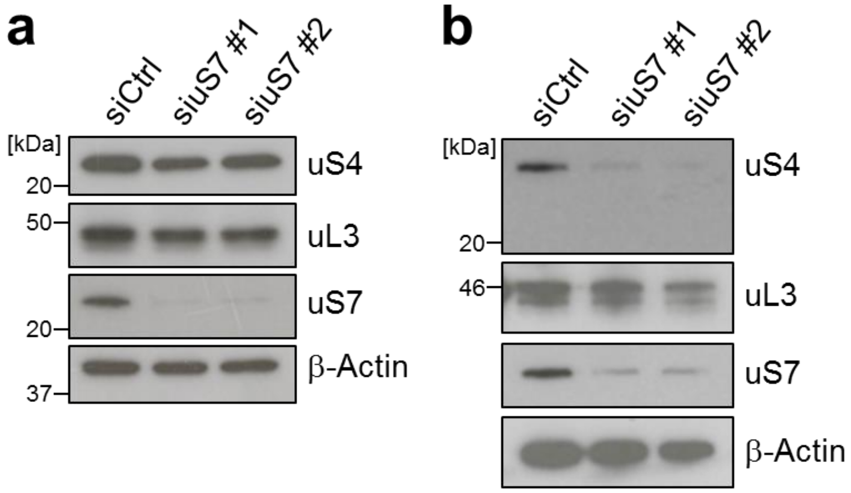

Supplementary Fig. 3 Effects of the knockdown of uS7 on other ribosomal proteins. HT-29 (**a**) and SW480 (**b**) cells were transfected with siCtrl, siuS7 #1, or siuS7 #2. After 48 h, cells were lysed and subjected to Western blotting.

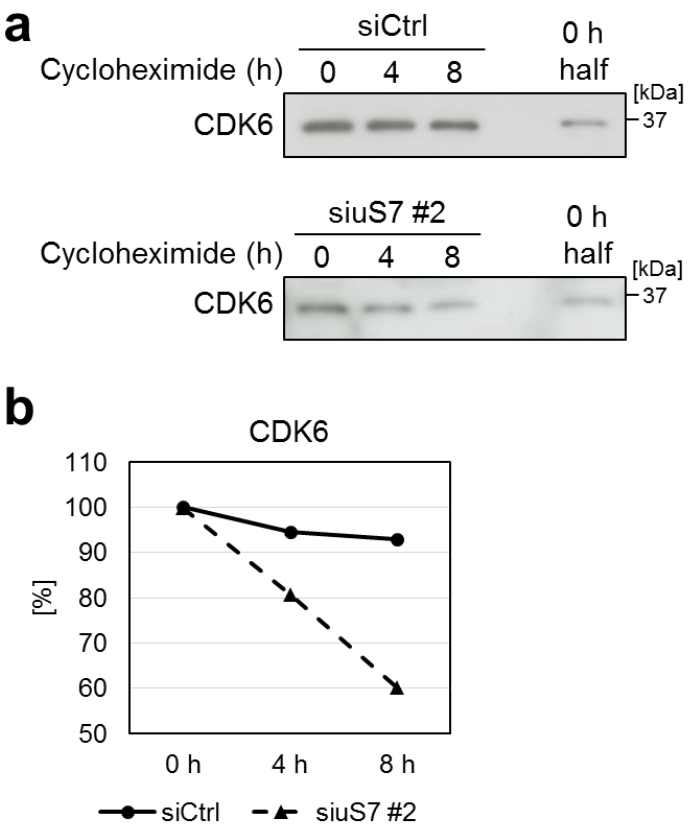

Supplementary Fig. 4 uS7 contributes to CDK6 stability in SW480 cells. (**a**) SW480 cells were transfected with siCtrl or siuS7 #2. After 18 h, cells were treated with 20 μg/ml cycloheximide and lysed at the indicated times. CDK6 protein expression levels were analyzed by Western blotting. The sample, designated as "0 h half", is identical to half the amount of the sample at 0 h. (**b**) CDK6 expression levels at each time point were quantified. The expression level at 0 h was defined as 100%.

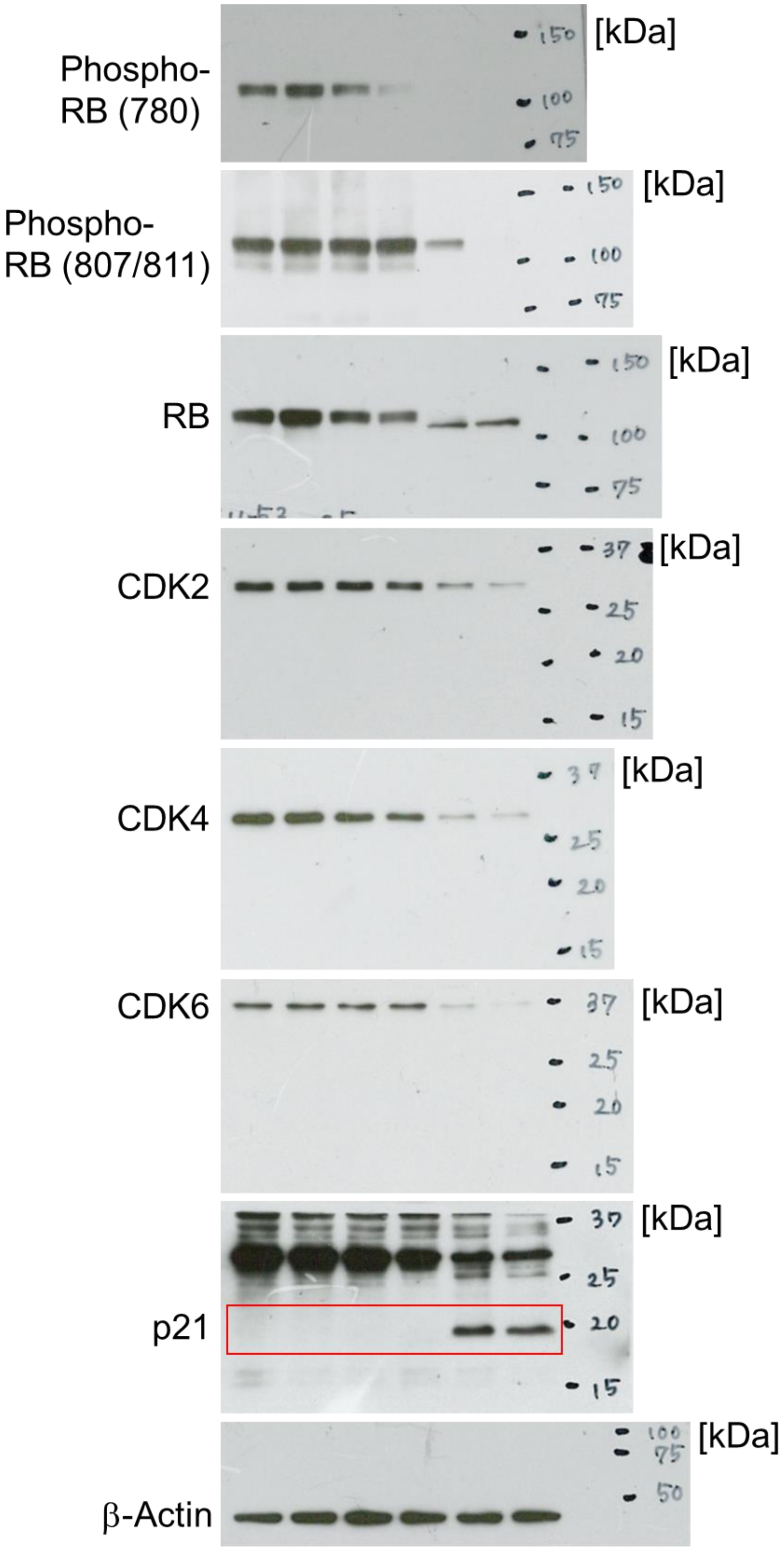

Supplementary Fig. 5   Uncropped Western blots of Fig. 1c.

Fig. 2d

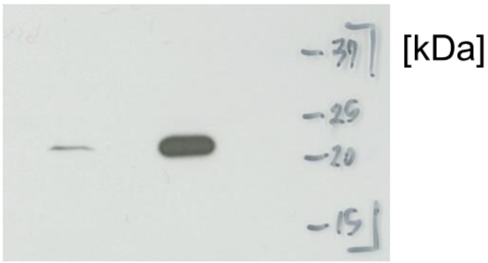

Fig. 2e

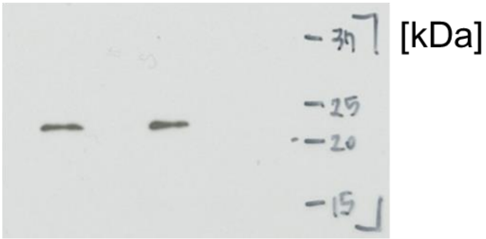

Supplementary Fig. 6   Uncropped Western blots of Fig. 2d and e.

Fig. 4a

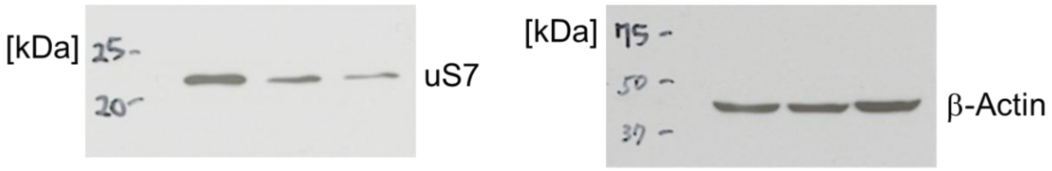

Fig. 4d

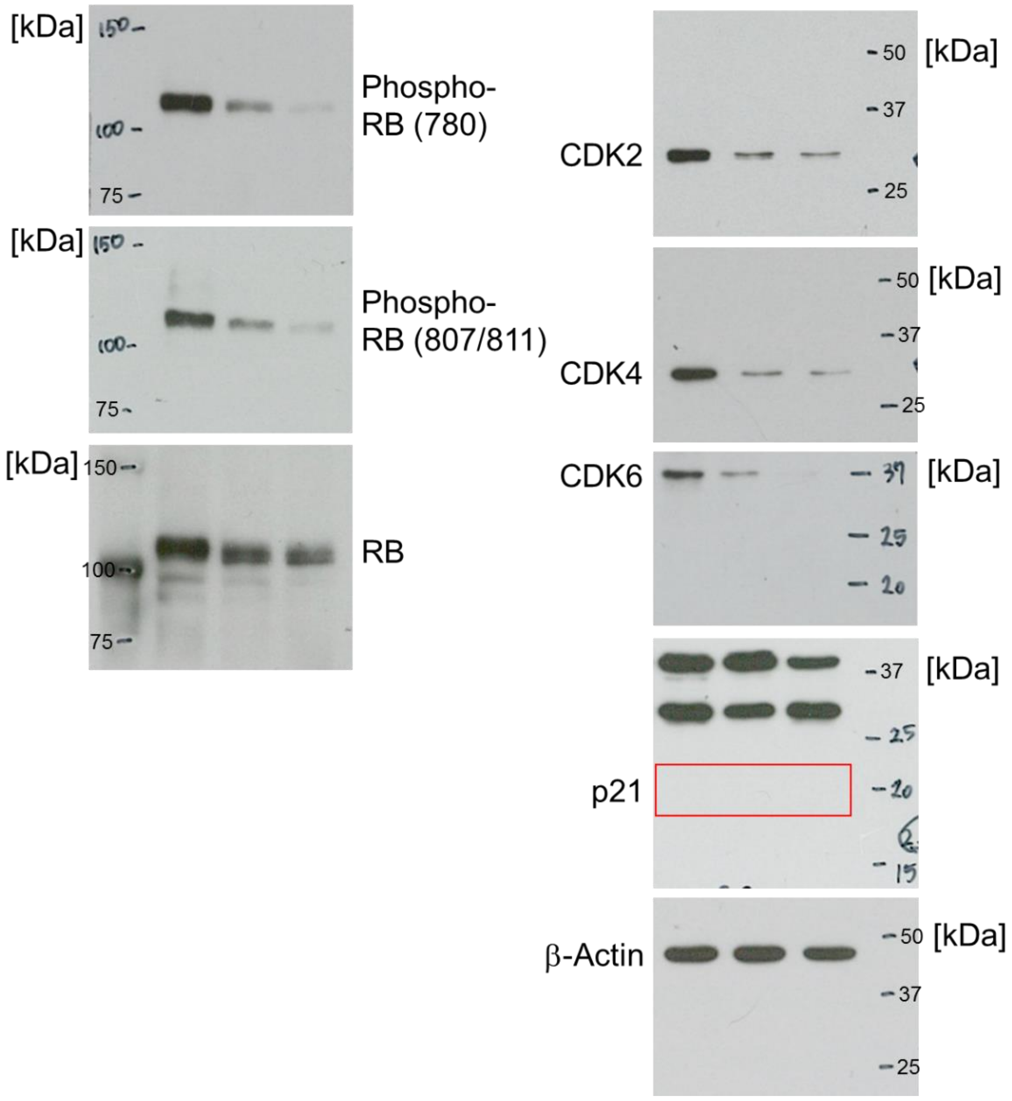

Supplementary Fig. 7　Uncropped Western blots of Fig. 4a and d.

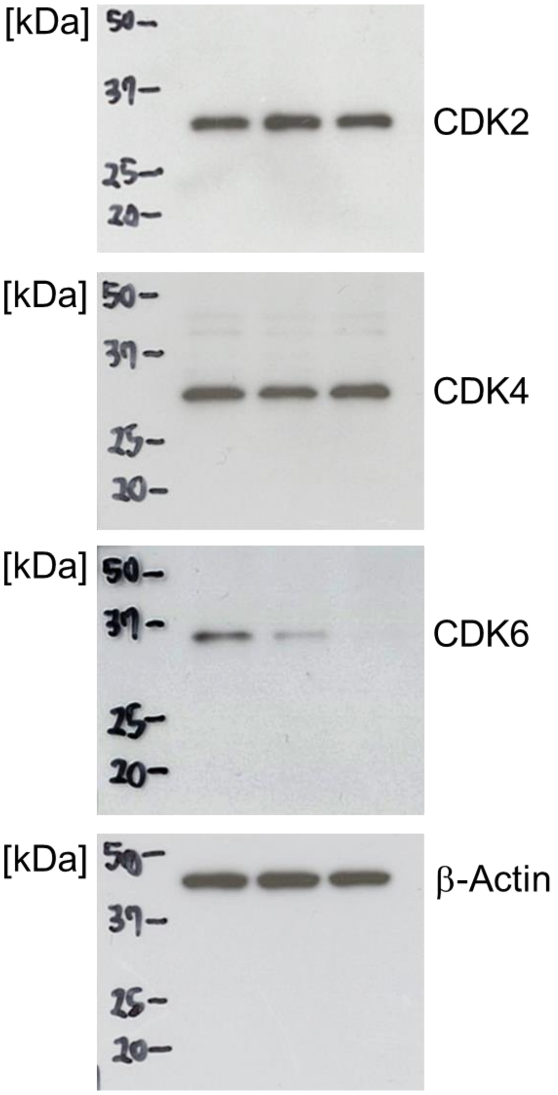

Supplementary Fig. 8 Uncropped Western blots of Fig. 4e.

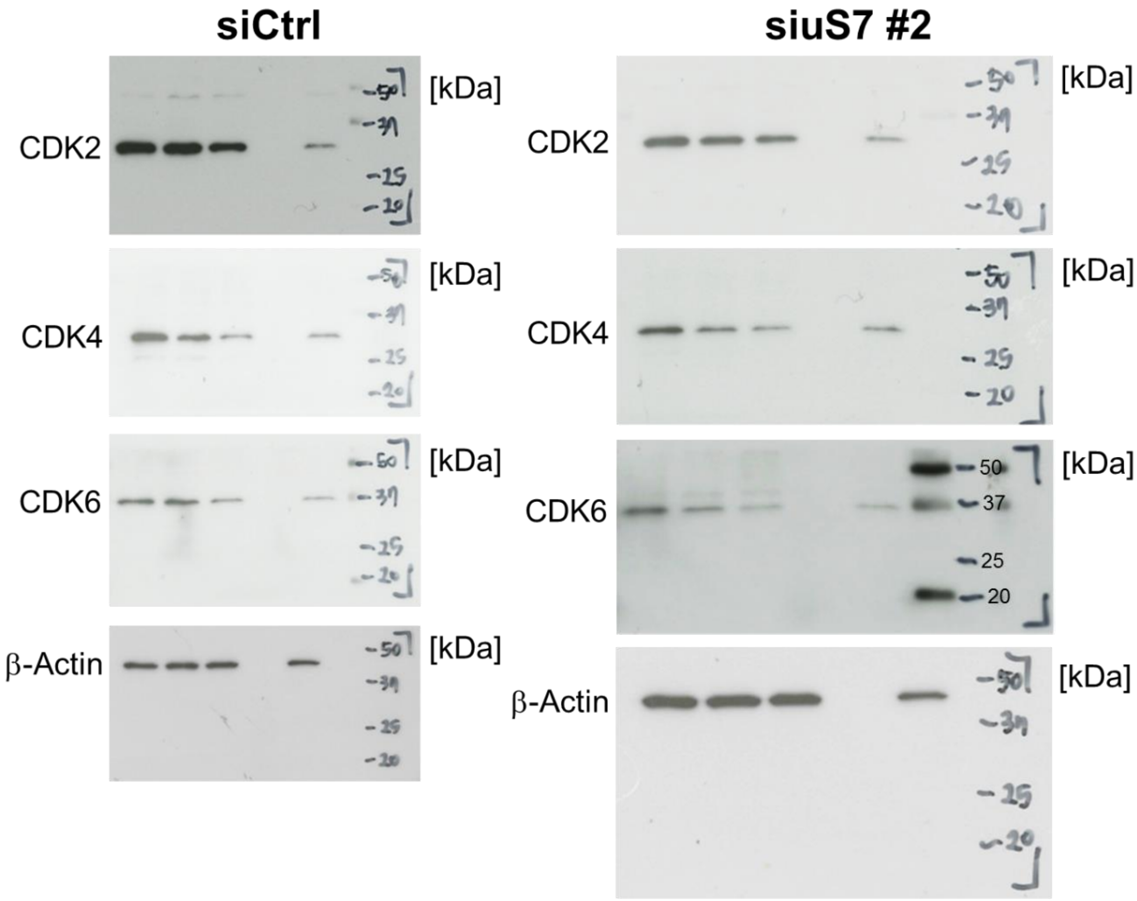

Supplementary Fig. 9   Uncropped Western blots of Fig. 5a.

Fig. 5c

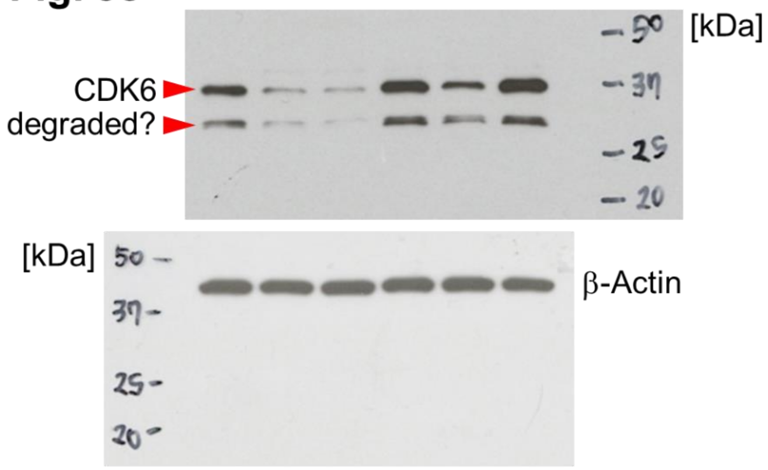

Fig. 5d

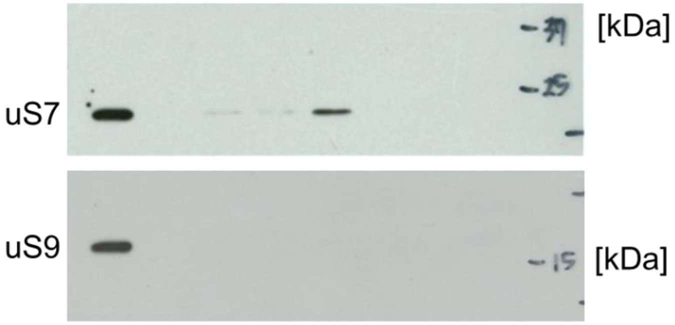

Fig. 5e

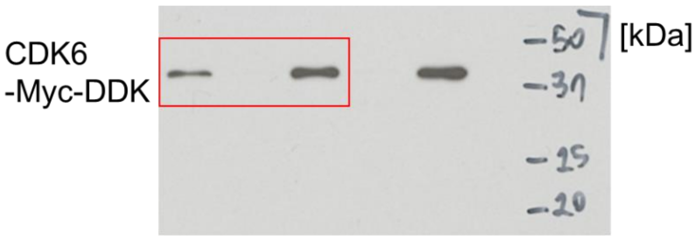

Supplementary Fig. 10 Uncropped Western blots of Fig. 5c-e.

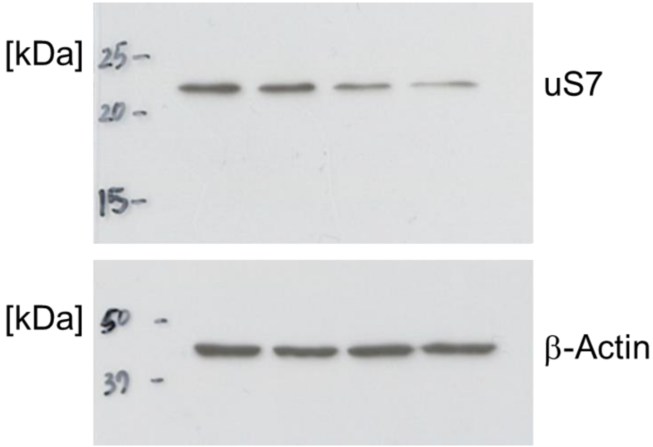

Supplementary Fig. 11   Uncropped Western blots of Fig. 6a.

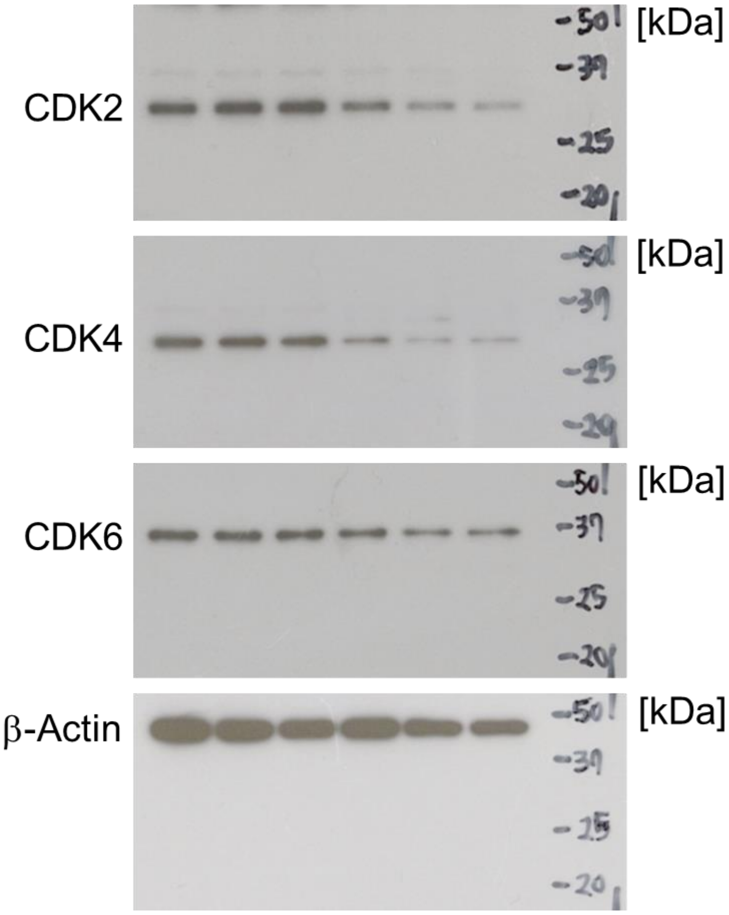

Supplementary Fig. 12 Uncropped Western blots of Supplementary Fig. 1c.

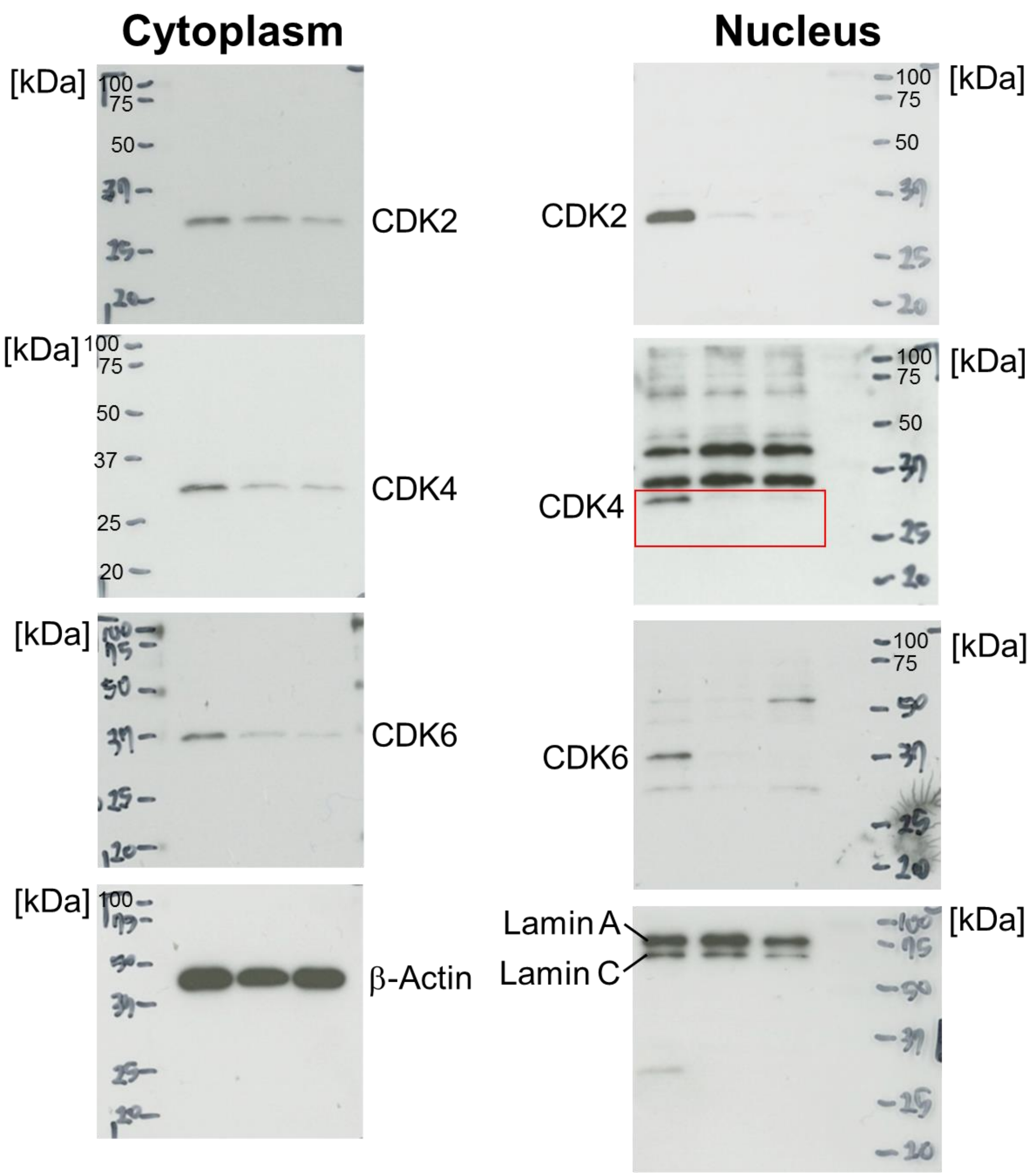

Supplementary Fig. 13 Uncropped Western blots of Supplementary Fig. 2.

Supplementary Fig. 3a

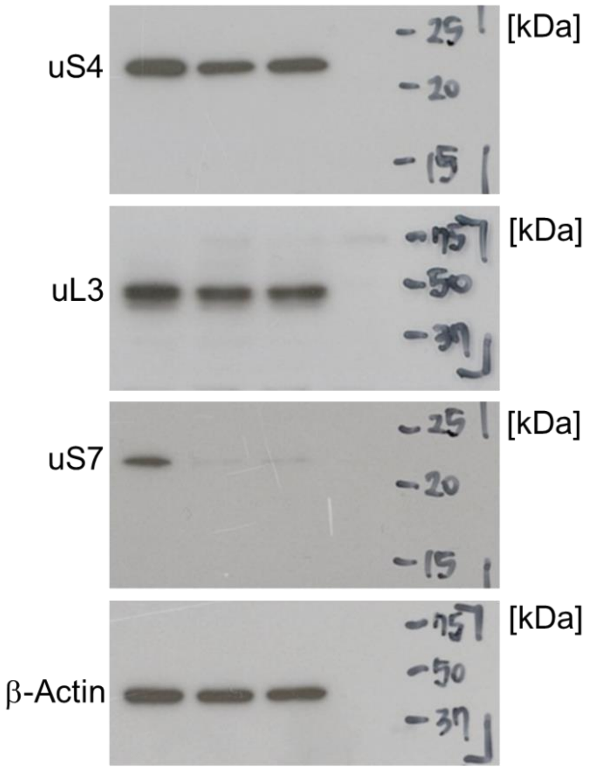

Supplementary Fig. 3b

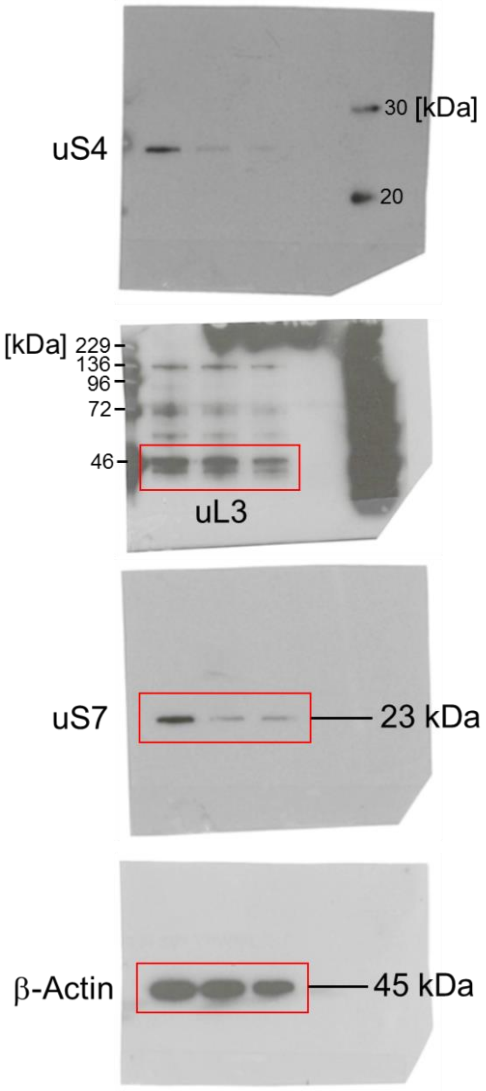

Supplementary Fig. 4a

siCtrl

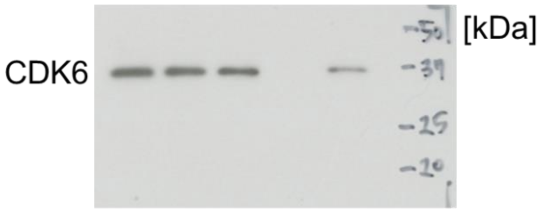

siuS7 #2

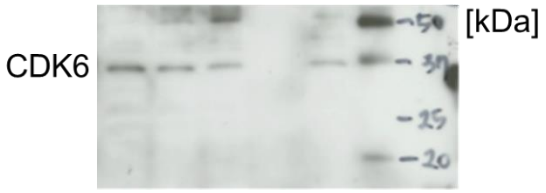

Supplementary Fig. 14 Uncropped Western blots of Supplementary Fig. 3 and 4.
