# Supplementary material for: Stabilization of CDK6 by ribosomal protein uS7, a target protein of the natural product fucoxanthinol
Source: Commun Biol. 2022 Jun 9;5:564. doi: 10.1038/s42003-022-03522-6 (PMC9184650; doi:10.1038/s42003-022-03522-6)
Supplement: Supplementary file 3 — Description of Additional Supplementary Files [file 42003_2022_3522_MOESM3_ESM.pdf]

Description of Additional Supplementary Files

File name: Supplementary Data 1
Description: The source data for the graphs in the main figures.

File name: Supplementary Data 2
Description: The charge of the fucoxanthinol molecule modeled using quantum chemical calculations. The charge is described below in the Tripos Mol2 format.

File name: Supplementary Movie 1
Description: The MD simulation trajectory of the uS7-fucoxanthinol complex (MP4 file). Fucoxanthinol (cyan stick) and uS7 (gray cartoon) are shown.
