# Supplementary material for: Stabilization of CDK6 by ribosomal protein uS7, a target protein of the natural product fucoxanthinol
Source: Commun Biol. 2022 Jun 9;5:564. doi: 10.1038/s42003-022-03522-6 (PMC9184650; doi:10.1038/s42003-022-03522-6)
Supplement: Supplementary file 7 — Reporting Summary [file 42003_2022_3522_MOESM7_ESM.pdf]

nature research

Corresponding author(s): Yosuke Iizumi

Last updated by author(s): May 2, 2022

Reporting Summary

Nature Research wishes to improve the reproducibility of the work that we publish. This form provides structure for consistency and transparency in reporting. For further information on Nature Research policies, see our Editorial Policies and the Editorial Policy Checklist.

Statistics

For all statistical analyses, confirm that the following items are present in the figure legend, table legend, main text, or Methods section.

n/a	Confirmed	
☐	☒	The exact sample size (*n*) for each experimental group/condition, given as a discrete number and unit of measurement
☐	☒	A statement on whether measurements were taken from distinct samples or whether the same sample was measured repeatedly
☐	☒	The statistical test(s) used AND whether they are one- or two-sided<br>*Only common tests should be described solely by name; describe more complex techniques in the Methods section.*
☒	☐	A description of all covariates tested
☒	☐	A description of any assumptions or corrections, such as tests of normality and adjustment for multiple comparisons
☐	☒	A full description of the statistical parameters including central tendency (e.g. means) or other basic estimates (e.g. regression coefficient) AND variation (e.g. standard deviation) or associated estimates of uncertainty (e.g. confidence intervals)
☐	☒	For null hypothesis testing, the test statistic (e.g. F, t, r) with confidence intervals, effect sizes, degrees of freedom and P value noted<br>*Give P values as exact values whenever suitable.*
☒	☐	For Bayesian analysis, information on the choice of priors and Markov chain Monte Carlo settings
☒	☐	For hierarchical and complex designs, identification of the appropriate level for tests and full reporting of outcomes
☒	☐	Estimates of effect sizes (e.g. Cohen's *d*, Pearson's *r*), indicating how they were calculated

Our web collection on statistics for biologists contains articles on many of the points above.

Software and code

Policy information about availability of computer code

Data collection	No special softwares were used.
Data analysis	Statistical analyses were performed using a non-repeated measures ANOVA followed by Bonferroni correction using the Excel Statistical Program File ystat2013 (Igakutosho Shuppan Ltd., Saitama, Japan).

For manuscripts utilizing custom algorithms or software that are central to the research but not yet described in published literature, software must be made available to editors and reviewers. We strongly encourage code deposition in a community repository (e.g. GitHub). See the Nature Research guidelines for submitting code & software for further information.

Data

Policy information about availability of data

All manuscripts must include a data availability statement. This statement should provide the following information, where applicable:

- Accession codes, unique identifiers, or web links for publicly available datasets
- A list of figures that have associated raw data
- A description of any restrictions on data availability

All data generated or analyzed during the present study are included in the article and its Supplementary Information file. Source data for the main figures can be found in Supplementary Data 1. All relevant data relating to the article are available from the corresponding author on reasonable request. Uncropped blots are provided as Supplementary Figures 5-14 in the Supplementary Information file.

Field-specific reporting

Please select the one below that is the best fit for your research. If you are not sure, read the appropriate sections before making your selection.

☒ Life sciences ☐ Behavioural & social sciences ☐ Ecological, evolutionary & environmental sciences

For a reference copy of the document with all sections, see nature.com/documents/nr-reporting-summary-flat.pdf

Life sciences study design

All studies must disclose on these points even when the disclosure is negative.

Sample size	At least independent triplicates were examined in all experiments. Sample sizes were determined based on generating convincing results.
Data exclusions	No data exclusions were needed.
Replication	All experiments were performed at least two times with similar results.
Randomization	This study does not contain experiments which require randomization.
Blinding	Blinding was not necessary in this study.

Reporting for specific materials, systems and methods

We require information from authors about some types of materials, experimental systems and methods used in many studies. Here, indicate whether each material, system or method listed is relevant to your study. If you are not sure if a list item applies to your research, read the appropriate section before selecting a response.

Materials & experimental systems

n/a	Involved in the study	
☐	☒	Antibodies
☐	☒	Eukaryotic cell lines
☒	☐	Palaeontology and archaeology
☒	☐	Animals and other organisms
☒	☐	Human research participants
☒	☐	Clinical data
☒	☐	Dual use research of concern

Methods

n/a	Involved in the study	
☒	☐	ChIP-seq
☐	☒	Flow cytometry
☒	☐	MRI-based neuroimaging

Antibodies

Antibodies used	The antibodies used in the present study were anti-phospho-RB-Ser780 (#9307, Cell Signaling Technology, Danvers, MA, USA), phospho-RB-Ser807/811 (#9308, Cell Signaling Technology), RB (Cat. No. 554136, BD Biosciences, San Jose, CA, USA), CDK2 (sc-163, Santa Cruz Biotechnology, Dallas, TX, USA), CDK4 (sc-601, Santa Cruz Biotechnology), CDK6 (sc-177, Santa Cruz Biotechnology), p21 (sc-397, Santa Cruz Biotechnology), β-actin (A5441, Sigma-Aldrich, St. Louis, MO, USA), Lamin A/C (#2032, Cell Signaling Technology), uS7 (ab58345 or ab210745, Abcam, Cambridge, UK), uS9 (ab26159, Abcam), uS4 (ab74711, Abcam), uL3 (ab241412, Abcam), cyclin D (Cat. No. 06-137, Merck KGaA, Darmstadt, Germany), cyclin E (sc-198, Santa Cruz Biotechnology), DDK (TA50011-100, OriGene Technologies, Rockville, MD, USA), HRP-linked anti-rabbit IgG (NA934V, GE Healthcare, Chicago, IL, USA), HRP-linked anti-mouse IgG (NA931V, GE Healthcare), and normal rabbit IgG (sc-2027, Santa Cruz Biotechnology).
Validation	These antibodies have been validated by each manufacturer.

Eukaryotic cell lines

Policy information about cell lines

Cell line source(s)	Human colon cancer cell lines HT-29 and SW480
Authentication	Authenticated by the National Cancer Institute Developmental Therapeutics Program or the American Type Culture Collection.
Mycoplasma contamination	All cell lines were negative for mycoplasma contamination.
Commonly misidentified lines (See ICLAC register)	This study does not contain commonly misidentified cell lines.

Flow Cytometry

Plots

Confirm that:

- [ ] The axis labels state the marker and fluorochrome used (e.g. CD4-FITC).
- [ ] The axis scales are clearly visible. Include numbers along axes only for bottom left plot of group (a 'group' is an analysis of identical markers).
- [ ] All plots are contour plots with outliers or pseudocolor plots.
- [ ] A numerical value for number of cells or percentage (with statistics) is provided.

Methodology

Sample preparation	Treated HT-29 or SW480 cells were harvested by trypsinization. After centrifugation at 780 × g for 5 min at room temperature, the cells were suspended in phosphate-buffered saline containing 0.1% Triton X-100, 150 µg/ml RNase A, and 5 µg/ml propidium iodide. The suspension was filtered through a mesh sheet (Kurabo Industries Ltd., Osaka, Japan).
Instrument	FACSCalibur (Becton, Dickinson and Company, Franklin Lakes, NJ, USA)
Software	BD CellQuest Pro software (version 6.0; Becton, Dickinson and Company, Franklin Lakes, NJ, USA) and ModFit LT software (version 3.3.11; Verity Software House, Inc., Topsham, ME, USA)
Cell population abundance	10,000 events were recorded for each sample.
Gating strategy	No gating was used in all experiments.

- [ ] Tick this box to confirm that a figure exemplifying the gating strategy is provided in the Supplementary Information.
